# Sputum smear grading and associated factors among bacteriologically confirmed pulmonary drug-resistant tuberculosis patients in Ethiopia

**DOI:** 10.1186/s12879-021-05933-y

**Published:** 2021-03-05

**Authors:** Getahun Molla Kassa, Mehari Woldemariam Merid, Atalay Goshu Muluneh, Dawit Tefera Fentie

**Affiliations:** 1 Department of Epidemiology and Biostatistics, Institute of Public Health, College of Medicine and Health Sciences, University of Gondar, Gondar, Ethiopia; 2 Gondar City Health Department, Amhara Regional State Health Bureau, Ministry of Health-Ethiopia, Gondar, Ethiopia

**Keywords:** Sputum-smear grading, Associated factors, Drug-resistant tuberculosis, Ethiopia

## Abstract

**Background:**

The sputum smear bacilliary load is a fundamental indicator of the level of infectiousness in DR-TB patients. However, evidence on DR-TB sputum smear grading and its factors in the study setting is limited. This study was aimed to determine the level of sputum smear grading and associated factors among DR-TB patients in Ethiopia.

**Methods:**

This was an institution based cross-sectional study on 520 bacteriological confirmed pulmonary DR-TB patients from September 2010 to December 2017 in the northwest Ethiopia. Epidata 4.2.00 and SPSS 20 were used for data entry and management, respectively. Ordinary logistic regression was fitted. A cut of *p*-value less than 0.05 in the multivariable ordinary logistic regression was considered to declare statistically significant variables.

**Results:**

Of all 520 bacteriological confirmed pulmonary DR-TB patients; 34.42% had 3^+^, 15.77% had 2^+^, 18.27% had 1^+^, 15.19% had scanty, and 16.35% had negative sputum smear grading results. The odds of having higher sputum smear grades were significantly associated with the patient’s educational status of secondary (Adjusted Odds Ratio (AOR) = 0.43, 95% Confidence Interval (CI): 0.21, 0.89), body mass index of 16 to 18.49 kg/m^2^ (AOR = 1.81, 95%CI: 1.16, 2.84), and TB treatment history of two and more times (AOR = 1.78, 95%CI: 1.24, 2.55).

**Conclusions:**

More than a third of the bacteriological confirmed pulmonary DR-TB patients in the study setting was highly infectious with the highest bacillary load. The odds of having a high bacillary load were significantly associated with the patient’s TB treatment history, nutritional, and educational status.

## Background

Tuberculosis (TB) caused by *Mycobacterium tuberculosis (MTB*) bacteria is ranked as the leading cause of death among infectious diseases in human history [1]. It claimed over a billion lives in the past two centuries [2]. The World Health Organization (WHO) declares TB as a global public health emergency in 1993. And set different TB care and control strategies and targets to create a TB-free world by the year 2035 [3]. Drug-Resistant Tuberculosis (DR-TB) continues to be one of the challenging global public health threats with more than half a million new cases reported annually in the year 2018 [1, 4]. Respectively, the global prevalence of DR-TB was 18 and 3.4% among the previously treated and new TB cases. While in Ethiopia, the estimated DR-TB prevalence was 0.71% among new cases and 16% of previously treated cases [1]. According to the 2017/2018 Ethiopian national TB reference laboratory data, the proportion of Multidrug-Resistant Tuberculosis (MDR-TB) was 11.6% [5]. The burden of DR-TB poses further challenges to the TB control program in Ethiopia.

Sputum Acid Fast Bacilli (AFB) positive pulmonary tuberculosis is the infectious form of tuberculosis and mainly responsible for transmitting the disease [6]. Through AFB staining, it is possible to quantify the mycobacterial load in the sputum. Accordingly, sputum smear is graded as scanty, 1+, 2+, and 3+. Scanty is when the sputum contains 1–9 AFB in 100 fields, grade 1+ for 10–99 AFB in 100 fields, grade 2+ if 1–10 AFB per field (check 50 fields), and grade 3+ for more than 10 AFB per field (check 20 fields), respectively [7].

Infectiousness of tuberculosis correlates with the number of bacilli in the sputum which is expressed by the sputum smear grading and the length of infectiousness. It is evidenced that patients with higher sputum AFB grade could have a higher chance of transmitting the disease and developing active TB among contacts than patients with lower grading [8]. Higher-order smear grading is associated with poor treatment outcome and increased mortality rate due to TB [9]. Thus, patients with high sputum AFB grade are a potential source of continued TB spread and a nexus for further drug resistance TB development in the community. As a result, it will pose a significant challenge in TB prevention and control strategy.

However, to the best of our search, there is no literature about sputum AFB grading and associated factors. Thus, we aimed to determine the level of sputum smear grading and associated factors among bacteriologically confirmed pulmonary DR-TB patients registered for second-line ant-TB treatment in Ethiopia.

## Methods

### Study design, setting, and population

A multicenter institution-based cross-sectional study was conducted from September 2010 to December 2017 among bacteriologically confirmed pulmonary DR-TB patients in the four DR-TB Treatment Initiating Centers (TICs) of Amhara regional state of Ethiopia. Amhara region is the second populated region in Ethiopia which contributes to the highest number of DR-TB in the country. A total of nine treatment initiating center were available in the Amhara regional state during the study period. From those nine TICs, the University of Gondar Hospital, Borumeda Hospital, Debre-Markos Hospital, and Woldia Hospital were selected for the study. These TICs were the oldest in the region, which contributes 90% of the DR-TB burden in the region. All the selected four TIC was evenly distributed in all corners of the region. These four TICs were giving DR-TB diagnosis and treatment services not only to the Amhara region which they exist but also to the nearest region around them including Tigray, Afar, and Benishangul-Gumuz regional populations. The source population for the study was all the DR-TB patients in the Amhara region and the study population was all bacteriologically confirmed pulmonary DR-TB patients in the selected four TICs. In this study, the DR-TB patient was defined as a patient with a bacteriologically confirmed result resistant to one of these; rifampicin resistant (RR), MDR, or Pre-XDR) as confirmed by using either of the Xpert/MTB Rif test or Line Probe Assay (LPA) test or solid/liquid culture media.

### Data collection and quality control

The sources of data were patients’ medical charts and unit TB registration books. The data was collected using a pretested data extraction checklist. Before extracting data from records, data collectors were practically trained and instructed to check the completeness and reliability of each data before submission. The quality of the data collection process and the completeness of the collected data were cheeked and supervised daily.

### Variables of the study and measurement

The dependent variable in this research was the level of sputum smear grading. After education and demonstration of patients on how to give quality sputum, three sputum samples (spot-morning-spot) were collected from all patients before they start ant-TB treatment. All the sputum were stained for acid-fast bacilli (AFB) and examined using a 100 times magnified objective lens of the Ziehl-Neelsen (ZL) method. The degree of sputum AFB positivity or grading was assigned to one of the five categories (negative, scanty, 1^+^, 2^+^, and 3^+^). The sputum bacillary load of was graded as negative when there were no tuberculosis bacilli per 100 field of observation, as scanty when there was one to nine bacilli per 100 field of observation, as plus one (1+) when there was ten to ninety-nine bacilli per 100 fields of observations, plus two (2+) when the observed bacilli were one to ten per field, and three plus (3+) when the number of bacilli was greater than ten per field of observation. The independent variables for this study were patients’ sociodemographic, behavioral, and clinical characteristics. Patients sex (categorized as male and female), age in years (ordered as less than 25, 25 to 44, and above 44), residency (categorized as urban and rural), educational status (ordered as no formal education, primary, secondary, and certificate and above), occupation (categorized as private business for those who had and run their own business, employed for those who had employed at the governmental or none governmental institutions, and unemployed for those individual with no job or no continues source of money), marital status (categorized as married, never married, divorced/widowed/separated), housing condition (dichotomized as homeless and having home, those patients who live in the street or lacks permanent night sleeping area were labeled as homeless), treatment supporter (dichotomized as yes or no), history of cigarette smoking (characterized as smoker and none smoker, we say smoker when the patient has a documented history of cigarettes smoking in their medical chart irrespective of the dose and duration), alcohol drinking (labeled as yes or no, patients who had a documented history of alcohol intake in their chart were grouped as yes), and khat chewing (grouped as yes or no, khat was a stimulant substance and patients was labeled as chewer when they have a documented history of khat chewing in their medical history) were the used sociodemographic and behavioral variables, while patients functional status (classified as working, ambulatory, and bedridden), anatomical site of tuberculosis (dichotomized as pulmonary and disseminated, when the TB affects only the lung parenchyma we consider as pulmonary but if it involves both the lung parenchyma and any other site we classified as disseminated), comorbidity (labeled as yes or no), Human Immunodeficiency Virus (HIV) coinfection (dichotomized as positive or negative), Body Mass Index (BMI) (ordered as < 16 kg/m^2^, 16 to 18.49 kg/m^2^, and > 18.5 k gram per meter square (kg/m^2^), which was calculated dividing patients weight in kilo gram to patients high in meter square), and the number of tuberculosis treatment history was the used as clinical variables.

### Data processing and statistical analysis

Data was entered into Epi-data 4.2.0.0 to minimize entry error and exported to SPSS 20 statistical software for further data management and analysis. Frequency and percent were used to describe discrete variables whereas we used to mean with Standard Deviation (SD) for normally distributed and median with Interquartile Range (IQR) for skewed continuous data. The bivariable and multivariable ordinary logistic regression model was fitted. The assumption in ordinary logistic regression (proportional odds) was checked at − 2 Log-Likelihood (−2LL) by using the Chi-Square test. The null hypothesis in the proportional odds assumption stated that the effects of any explanatory variables are the same across response categories. We declare that the assumption is satisfied when the test statistics for proportional odds has a *p*-value of > 0.05. In the case of this study, the p-value for the proportional odds assumption test was 0.3222 and we accept the null hypothesis. The pseudo R^2^ values were determined to compare whether the fitted model with the explanatory variables significantly predicts the outcomes than the intercept at -2LL. The final model goodness-of-fit was assessed by using Pearson’s and deviance Chi-square statistics. These chi-squared statistics are intended to test whether the observed data are consistent with the fitted model. The null hypothesis was the model is fit while the alternative hypothesis was the model is not fit. We accept the null hypothesis when the Pearson’s and deviance chi-square statistics has a *p*-value of above 0.05. In this study, the *p*-value of Pearson’s and deviance chi-square statistics was 0.582 and 0.990, respectively. Those variables with a p-value of less than 0.2 in the bivariable model were fitted in the multivariable model and considered statistically significant when the p-value was less than 0.05.

## Results

### Socio-demographic and behavioral characteristics

Of the total 529 registered bacteriologically confirmed pulmonary DR-TB patients in the study area during the study period, we analyzed the data of 520 patients. Because the data related to smear grading for 9 patients were missed. More than half (56.92%) were males. The median and SD of age was 31.88 + 12.18 years and above a half (52.12%) were in the age group of 25 to 44 years old. One-third of the study participants were single in their marital status (Table 2).

### Clinical characteristics

Nearly two-thirds (64.67%) of patients had a poor functional status (ambulatory and bedridden). More than a quarter were HIV co-infected (27.75%) and three-fourth (75.66%) were malnourished with a BMI of less than 18.5 Kg/m^2^. Most of the patients have a history of repeated first-line anti-TB treatments (Table 1).

**Table 1:** Clinical characteristics of bacteriological confirmed pulmonary DR-TB patients (*N* = 520)

Variables	Categories	Frequency (percent)	Smear grading of DR-TB				
			**0**	**1–9**	**1**^**+**^	**2**^**+**^	**3**^**+**^
Functional status	Working	183 (35.33)	33	29	37	23	61
	Ambulatory	247 (47.68)	33	42	52	40	80
	Bedridden	88 (16.99)	19	7	6	18	38
Anatomical site of tuberculosis	Pulmonary	500 (96.15)	80	77	93	78	172
	Disseminated	20 (3.85)	5	2	2	4	7
Comorbid disease	Yes	78 (15.00)	10	12	10	16	30
	No	442 (85.00)	75	67	85	66	149
HIV^a^ co-infection	Yes	144 (27.75)	27	23	17	16	61
	No	375 (72.25)	58	56	77	66	118
Body mass index Kg/M^2^	< 16	217 (42.14)	42	32	32	31	80
	16–18.49	171 (33.20)	17	22	37	33	62
	> = 18.5	127 (24.66)	24	25	24	17	37
Number of TB treatment history	< 2	249 (47.88)	51	44	42	33	79
	> = 2	271 (52.12)	34	35	53	49	100

^a^ *HIV* Human Immunodeficiency Virus

**Fig. 1:**
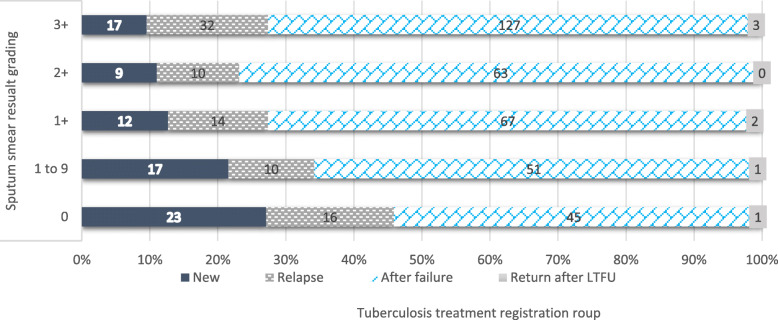
Distribution of sputum smear grading on the registration group among bacteriological confirmed pulmonary DR-TB patients (*N* = 520)

Most of the patients have a TB registration group of failure to first-line ant-TB treatment. Similarly, those patients with smear grading of 3+ were patients registered as a failure of first-line TB treatment. The magnitude of primary DR-TB was also high **(**Fig. 1**).**

### The level of sputum smear grading of DR-TB

Of all 520 pulmonary DR-TB patients, 16.35% (95% CI: 13.40, 19.79) were sputum smear-negative. The rest 15.19% (95% CI: 13.40, 19.79) had scanty (1 to 9 bacilli), 18.27% (95% CI: 12.35, 18.55) had a 1^+^, 15.77% (95% CI: 12.87, 19.17) had a 2^+^, and 34.42% (95% CI: 30.45, 38.65) had a 3^+^ sputum smear grading result. The overall magnitude of sputum smear positivity was 83.65% (95% CI: 80.21, 86.60).

### Factors associated with sputum smear grading

Those variables that satisfied the proportional odds assumption were fitted in the model. In the bivariable ordinary logistic regression model, those patients with treatment supporter, cigarette smokers, khat chewers, low body mass index, and has two and more previous tuberculosis treatment history were positively associated with sputum smear grading. While, patients with low educational status, homeless, and private business and unemployed were negatively associated with the patient’s sputum smear grading with a *p*-value of less than 0.2. After controlling the confounding effect of occupation, housing status, treatment supporter, cigarette smoking, and khat chewing in the multivariable ordinary logistic regression model, the variable educational status, body mass index, and history of previous tuberculosis treatment were significantly associated with sputum smear grading at a p-value of less than 0.05. The odds of having higher sputum smear grades for secondary educations were nearly half (AOR = 0.43, 95% CI: 0.21, 0.89) to the odds for certificate and above. Those patients with BMI of 16 to 18.49 kg/m^2^ are approximately having two times (AOR = 1.81, 95%CI: 1.16, 2.84) odds of higher sputum smear grading than BMI of > 18.5 kg/m^2^. The odds of having higher sputum smear grade were nearly twofold (AOR = 1.78, 95%CI: 1.24, 2.55) in patients having a TB treatment history of two and more compared to less than two times history of TB treatment (Table 2).

**Table 2:** Bi-variable and multivariable ordinary logistic regression analysis of sputum smear grading among bacteriological confirmed pulmonary DR-TB patients (*N* = 520)

Variables	Categories	Frequency (%)	Smear grading of DR-TB					COR (95%CI)	AOR (95%CI)
			**0**	**1–9**	**1**^**+**^	**2**^**+**^	**3**^**+**^		
Sex	Male	296 (56.92)	40	53	56	44	103	1	
	Female	224 (43.08)	45	26	39	38	76	0.93 (0.68, 1.27)	
Age in year	= < 24	162 (31.15)	30	26	36	20	50	1	1
	25–44	271 (52.12)	38	42	41	50	100	1.39 (0.98, 1.96)	1.27 (0.87, 1.95)
	> = 45	87 (16.73)	17	11	18	12	29	1.10 (0.69, 1.75)	1.01 (0.56, 1.83)
Residence	Urban	276 (53.18)	47	40	50	45	94	1	
	Rural	243 (46.82)	38	39	44	37	85	1.03 (0.75, 1.39)	
Educational status	No formal education	232 (44.82)	45	33	39	34	81	0.57 (0.34, 0.94)	0.49 (0.23, 1.03)
	Primary	138 (26.74)	21	19	32	20	46	0.59 (0.35, 1.02)	0.51 (0.25, 1.05)
	Secondary	82 (15.89)	11	20	15	16	20	0.47 (0.26, 0.84)	**0.43 (0.21, 0.89)**
	Certificate and above	64 (12.40)	8	6	9	11	30	1	1
Occupation	Employed	55 (10.68)	9	5	8	10	23	1	1
	Private business	313 (60.78)	51	48	55	45	114	0.77 (0.46, 1.30)	1.40 (0.65, 3.03)
	Unemployed	147 (28.54)	25	26	29	27	40	0.61 (0.35, 1.07)	1.08 (0.49, 2.35)
Marital status	Married	250 (48.36)	43	36	42	46	83	1	
	Never Married	179 (34.62)	28	26	39	26	60	1.00 (0.71, 1.40)	
	Divorced/widowed/Separated	88 (17.02)	14	17	14	10	33	1.01 (0.65, 1.57)	
Housing condition	Homeless	26 (5.79)	5	7	4	3	7	0.62 (0.31, 1.27)	0.48 (0.22, 1.04)
	Has home	423 (94.21)	62	65	86	68	142	1	1
Has treatment supporter	Yes	454 (88.16)	77	68	85	72	152	1	1
	No	61 (11.84)	7	9	9	10	26	1.45 (0.89, 2.36)	1.27 (0.75, 2.14)
History of Cigarette smoking	Yes	73 (14.04)	7	12	7	14	33	1.71 (1.09, 2.70)	1.45 (0.77, 2.73)
	No	447 (85.96)	78	67	88	68	146	1	1
History of alcohol drinking	Yes	83 (16.02)	7	19	14	13	30	1.16 (0.77, 1.76)	
	No	435 (83.98)	78	60	81	68	148	1	
History of khat Chewing	Yes	39 (7.53)	3	7	3	6	20	2.01 (1.08, 3.71)	1.62 (0.67, 3.90)
	No	479 (92.47)	82	72	92	75	158	1	1
Comorbid disease	Yes	78 (15.00)	10	12	10	16	30	1.31 (0.85, 2.02)	
	No	442 (85.00)	75	67	85	66	149	1	
Body mass index (kg/m^2^)	< 16	217 (42.14)	42	32	32	31	80	1.29 (0.87, 1.92)	1.46 (0.94, 2.25)
	16–18.49	171 (33.20)	17	22	37	33	62	1.65 (1.10, 2.48)	**1.81 (1.16, 2.84)**
	> = 18.5	127 (24.66)	24	25	24	17	37	1	1
Number of TB Treatmant history	Less than two	249 (47.88)	51	44	42	33	79	1	1
	Two and more	271 (52.12)	34	35	53	49	100	1.51 (1.11, 2.05)	**1.78 (1.24, 2.55)**

*Test of parallel lines (logit): Chi-Square = 45.667 p-value = 0.322, Goodness of fit in the Chi-Square test: Pearson (p-value = 0.582) and Deviance (p-value = 0.990), and Model information (pseudo R*^*2*^*): p-value = 0.0266*

## Discussion

This study was conducted to assess the level of sputum smear grading and associated factors among drug-resistant pulmonary tuberculosis in northwest Ethiopia. The overall sputum smear positivity rate was 83.65% among bacteriologically confirmed pulmonary DR-TB patients. This infers that approximately 85% of pulmonary DR-TB patients in the study area can transmit the drug resistance MTB strain to their contacts. We also found that from these bacteriologically confirmed pulmonary DR-TB patients 16% and more than one-third (34.42%) of the study patients had a sputum smear grade level of 2^+^ and 3^+^, respectively. This magnitude was lower than findings from a study conducted in Mali where nearly two-third of MDR-TB patients had a 3+ bacillary load [10]. This variation might be due to the difference in the study populations; the study in Mali was based on hospital admitted MDR-TB patients while our study was based on all bacteriological confirmed pulmonary DR-TB patients. The magnitude of 3^+^ sputum smear results in this study was in agreement with the findings in India, reported as 39% of smear-positive TB patients have 3^+^ sputum smear grading [11]. As the evidence showed that those smear-positive patients were more infectious and their probability of infectiousness increases with increased sputum smear grading [12]. High sputum smear grading was established as a result of long treatment delay which will also directly linked to extensive transmission to the communities because of large MTB load and prolonged contacts [13]. Study results from Ethiopia, China, Vietnam, and India evidenced that high smear grading was also associated with delayed smear and culture conversion, longer isolation period, more intensive treatment, and poor interim and final treatment outcomes [14–17]. High smear grading was also associated with the occurrence of MDR-TB [18]. Having highly infectious patients may increase the burden of tuberculosis in the general community that can impose more challenges to achieve End-TB strategies [19].

Higher sputum smear grading was significantly associated with the number of previous tuberculosis treatment, body mass index, and patient’s educational status in the multivariable ordinary logistic regression analysis.

The odds of having high sputum smear grade was nearly double among those patients with two and above previous TB treatment history compared to their counterparts. Tuberculosis recurrence was happened due to the TB chronicity, the presence of tick and multiple lung cavities, extensive lung parenchymal involvement, and presence of comorbid diseases; these pathological changes were also linked to high sputum smear grading. Evidence from work of literature showed that sputum smear grading was associated with TB recurrence and vice versa [20, 21]. In this study, more than half (52.12%) of patients had two and more and 85% had at least one episode of TB treatment history. From all, 353 (67.88%) and 82 (15.77%) were having a tuberculosis registration group of treatment failure and relapse respectively based on their most recent TB treatment history (Fig. 1).

This study revealed that a body mass index of 16 to 18.49 kg/m^2^ is positively associated with increase sputum smear grading. Evidence showed that the severity of BMI and the extent of affected lung area were linearly connected. Similarly, the degree of the affected lung was also directly correlated with a load of MTB bacilli in the sputum [22]. A study from Indonesia and Lithuania also indicated that underweight was associated with persistent sputum positivity [23, 24]. From this study, patients with the BMI categories of 16 to 18.48 kg/m2 were more alcoholic and cigarette smokers compared to patients with normal BMI. These behavioral characteristics were directly suppressing the immune response of patients and increased the burden of sputum tuberculosis bacillary load [24].

The odds of having higher sputum smear grades for secondary educations were nearly half compared to the odds for certificate and above. This might be due to the quality of sputum productions. We have observed from our clinical work that those patients with low educational status were negligent to internalize the health education given to produce quality sputum.

## Limitations

Due to the secondary nature of the data we were unable to see the association of the sputum smear grading with some important variables including radiological findings, time to smear, and culture conversions.

## Conclusion

Most of the DR-TB patients in the study setting were highly infectious and high bacillary load was significantly associated with the patient’s TB treatment history, nutritional, and educational status.

## Data Availability

The data used for the current study will be available based on a reasonable request from the lead author (Mr Getahun Molla Kassa).

## Abbreviations

DR-TB: Drug-Resistant Tuberculosis
TB: Tuberculosis
MTB: *Mycobacterium tuberculosis*
*MDR-TB*: Multidrug-Resistant Tuberculosis
WHO: World Health Organization
AOR: Adjusted Odds Ratio
CI: Confidence Interval
AFB: Acid-fast staining
ZL: Ziehl-Neelsen
TICs: Treatment Initiating Centers
LPA: Line Probe Assay
IQR: Interquartile Range
SD: Standard Deviation
-2LL: -2 Log-Likelihood
HIV: Human Immunodeficiency Virus
BMI: Body Mass Index (BMI), and kilogram per meter square (kg/m^2^)
